# Could sex-specific subtypes of hand osteoarthritis exist? A retrospective study in women presenting to secondary care

**DOI:** 10.3389/fpain.2024.1331187

**Published:** 2024-02-12

**Authors:** Malvika Gulati, Gretchen Brewer, Andrew Judge, Donna Kennedy, Tonia L. Vincent, Fiona E. Watt

**Affiliations:** ^1^ Centre for Osteoarthritis Pathogenesis Versus Arthritis, Kennedy Institute of Rheumatology, Nuffield Department of Orthopaedics, Rheumatology and Musculoskeletal Sciences (NDORMS), University of Oxford, Oxford, United Kingdom; ^2^ Department of Rheumatology, Charing Cross Hospital, Imperial College Healthcare NHS Trust, London, United Kingdom; ^3^ Musculoskeletal Research Unit, University of Bristol, Bristol, United Kingdom; ^4^ Centre for Statistics in Medicine, Nuffield Department of Orthopaedics, Rheumatology and Musculoskeletal Sciences, University of Oxford, Oxford, United Kingdom; ^5^ National Institute for Health Research Bristol Biomedical Research Centre (NIHR Bristol BRC), University Hospitals Bristol and Weston NHS Foundation Trust, Bristol, United Kingdom; ^6^ Therapies Department, Imperial College Healthcare NHS Trust, London, United Kingdom; ^7^ Department of Immunology & Inflammation, Hammersmith Campus, Imperial College London, London, United Kingdom

**Keywords:** phenotype, menopause, hormone replacement therapy (HRT), estrogen, female

## Abstract

**Introduction:**

Hand osteoarthritis is more common in women, and its risk increases around the time of the menopause. We set out to describe the timing between menopause and the onset of symptomatic hand osteoarthritis (OA), and associations with the use of hormone replacement therapy (HRT) or its discontinuation, describing any identifiable subgroups of women.

**Methods:**

Retrospective healthcare-records study of sequential women referred to a specialist hand OA clinic, 2007–2015. Confirmation of hand OA diagnosis was by clinican, by accepted criteria. Demographics and clinical variables were from healthcare-records, recorded by standardised proforma. Outcomes of interest were reported age of onset of hand symptoms, reported age at final menstrual period (FMP), time from FMP to reported onset of hand symptoms and time from cessation of HRT to reported onset of hand symptoms. Exposure categories for systemic HRT use were never users, current users, previous users. Analysis of Variance compared groups; linear regression analysed associations of exposure with outcome.

**Results:**

82/92(89%) of eligible women were post-menopausal, mean age at FMP 49.9 years (SD5.4). In these post-menopausal women, median time from FMP to hand symptom onset was 3 years. 48/82 (59%) developed hand symptoms within the defined peri-menopausal period (FMP ± 4 years), whilst some women developed their symptoms before or after (range −25, 30 years). In women who discontinued HRT prior to symptom onset, the median time from HRT cessation to onset of hand symptoms was 6 months. Past HRT users were older at hand symptom onset than women who had not taken HRT [coeff.4.7 years (0.92, 8.39); *P* = 0.015].

**Conclusions:**

This study adds to evidence associating the menopause/sex hormone deficiency with hand OA symptom onset in a sizeable subgroup of women (but not all). HRT use/cessation appears to influence the timing of onset of hand OA symptoms. It is not possible to interpret from this type of study whether sex hormone deficiency is causative of disease or modulates its symptoms. It is also not possible to judge whether painful hand osteoarthritis in post-menopausal women is a subtype of disease. Further investigation is indicated of sex-specific subtypes and potential for personalised medicine for post-menopausal women with hand osteoarthritis, as a clearly definable high-risk subgroup.

## Introduction

Osteoarthritis (OA) is in the top 20 leading causes of years lived with disability worldwide (1, 2). Hand OA is the commonest form, with symptoms affecting 47% of women and 25% of men over their lifetime (3). Of those over 45 years of age seeking treatment for hand OA, a large proportion are women [UK data, estimated 1.06 million (68%) (4)]. The disease can involve the interphalangeal joints (IPJs), the first carpometacarpal joint (CMCJ) or both, causing pain, deformity and functional impairment (3). Affected individuals have substantial impairment of quality of life which can be akin to rheumatoid arthritis (5). Those with symptomatic hand OA are more likely to experience cardiovascular disease (6). Evidence-based interventions for IPJ disease are limited and most commonly involve pain relief and exercise (7). There remains a poor understanding of disease pathogenesis of OA in general and hand OA in particular, with no pharmacological disease-modifying therapies for this disease, despite a number of recent clinical trials (8–10).

The apparent association between female sex, menopause and OA has long been noted (11). More recently, there has been growing circumstantial evidence for an association of OA with female sex: an increased risk in women in hand, hip and knee OA which becomes evident later in life is now well-documented (12–14). An upsurge in the relative risk of hand OA in women compared with men occurred around the age of 50 in a large Spanish study of electronic health records, the conserved typical age of menopause worldwide (14). In one Italian study in secondary specialist care, 90% of patients referred for “inflammatory”, erosive painful hand OA were women (15). In a large epidemiological study examining the association between hormone replacement therapy (HRT) and incident hand OA (using UK Community Practice Research Datalink data), current HRT use appeared protective when initiated around the time of the menopause, but this benefit diminished after discontinuing HRT (16).

However, there is no evidence for a direct association between the onset of hand OA and onset of menopause or direct evidence in humans for a causal relationship between the two. Whether the sex-specific epidemiology of this disease mean that either hand osteoarthritis in women or hand osteoarthritis in post-menopausal women might represent clinically relevant subgroups for study or personalised treatment has been inadequately explored.

Estrogen, or rather its deficiency, would appear to be important in the pathogenesis of OA and musculoskeletal pain. In animal models of surgically-induced knee OA, young female animals are protected from the disease compared with males, but lose this protection after ovariectomy (17). However several epidemiological studies examining the use of estrogen-containing HRT and its association with hand pain report conflicting findings (18–20). These studies are likely limited by substantial residual confounding (given that pain may be a reason to seek HRT prescription) and lack of validation cohorts (21). HRT use has been reported to have a significant protective effect on knee OA in Framingham cohort women (OR 0.31; 95% CI 0.11, 0.93) (22) and also in the Chingford cohort in the UK, where a weaker effect on hand joints was seen (23). Those in the unopposed estrogen arm of the Women's Health Initiative studies had reduced rates of total hip and knee replacement (24) and reduced musculoskeletal (MSK) pain of any cause (25). A systematic review found evidence for a protective effect of exogenous estrogen on hip OA (26). However there are very few randomized controlled trials (RCTs) testing the effects of estrogen-containing therapies in women included for MSK painful conditions (21). Only one study has been in symptomatic hand OA to our knowledge, which was a feasibility study so not designed to test efficacy (27).

We hypothesised that hand osteoarthritis in women around the time of menopause may represent an important phenotype of hand osteoarthritis. Within this phenotype, the onset of peri-menopause and menopause could influence the timing of onset of hand OA or its clinical presentation in the group as a whole, or in a subgroup of these women. Furthermore, use of HRT might also be associated with modified timing of hand symptom onset. Our aim was to explore these questions in a population of women with painful hand OA referred to a secondary care setting because of their hand symptoms, where relevant data were routinely collected. Our objectives were: (i) to describe the timing between the onset of menopause and the onset of hand OA symptoms; (ii) to explore if current or previous use of HRT was associated with the timing of onset of hand OA symptoms; (iii) to examine if cessation of HRT was associated with timing of onset of hand OA symptoms.

## Materials and methods

### Ethical approvals

Ethical approval was given for the analysis of usual care health records by the Sponsor Imperial College Healthcare NHS Trust in the UK for the purposes of this study (IRAS reference number 282499, REC 20/HRA/4630). The study conforms to STROBE guidelines (checklist in Supplementary Material) and included a pre-defined protocol for analysis which was reviewed as part of the ethical approval process.

### Patients and setting

A retrospective study of individual healthcare records was carried out using the records of consecutive new patients attending a multidisciplinary specialist hand OA clinic at Charing Cross Hospital [Imperial College Healthcare National Health Service (NHS) Trust, London, UK].

### Eligibility

Sequential records of those seen at the clinic as new patients for 8 years between 1 July 2007 and 30 June 2015 were reviewed and included if they were women aged 18 years and older and were diagnosed with hand OA by a rheumatologist. This diagnosis was in line with the American College of Rheumatology (ACR) definition of hand OA (28), considering clinical features including frequent hand joint pain with or without loss of function and consistent radiographic changes. Any case of hand OA was included (base of thumb OA only, interphalangeal joint OA only or having both sites affected). Those with other forms of arthritis (such as rheumatoid arthritis, gout or psoriatic arthritis), or other causes of hand pain as the main cause for pain (such as carpal tunnel syndrome or tenosynovitis) were excluded. Those using anti-estrogen treatments or chemotherapy were also excluded.

### Data

These were extracted from a standardised proforma which had been completed at all initial consultations during this period which recorded medical and medication history, including specific questions on reported age of final menstrual period (FMP) and age at onset of hand OA symptoms. Patients were considered post-menopausal if their FMP was at least 1 year previous, in the absence of hormonal contraception (29, 30). In the case of surgically induced menopause, age at surgery was used as age at FMP. We defined the peri-menopausal period for our study as 4 years before FMP and up to 4 years after FMP (31, 32). Available medication information included the use and timing of systemic (estrogen-containing) HRT (specifically, when it was started and stopped, or whether there was ongoing use). Based on this information, HRT use was further categorised as “Current”, “Previous” or “Never” (16, 30). “Current” HRT use was a minimum of 6 months of use, taken within the last 12 months; “Previous” use was defined as minimum of 6 months of use, with cessation more than 12 months ago. “Never” use was defined as no use, or less than 6 months of use (pre-menopausal females were also included in this group). Missing data were recorded as such. Participants were not excluded due to missing data.

### Analysis

All available data on all patients fulfilling criteria were analysed.

Predefined outcomes of interest were continuous variables of: (a) reported age of onset of hand symptoms; (b) reported age of FMP; (c) time from FMP to reported onset of hand symptoms; (d) time from cessation of HRT to reported onset of hand symptoms.

The main exposure of interest was use of systemic HRT (where groups were never users of HRT, current users and previous users).

One way Analysis of Variance (ANOVA) was used to compare the outcome, age of onset of hand symptoms (in years) between these 3 groups: those who had never used HRT, current users and previous users.

In addition, linear regression modelling was used to describe associations of HRT exposure groups with outcome, the age of onset of hand symptoms, in years. Effect size (regression coefficient of the difference in mean age at onset between groups, and its 95% confidence interval) therefore indicated the effect on additional years of age at onset of hand symptoms in each of two groups (previous users and current users) when compared with age of onset of hand symptoms in the reference group (never users of HRT). Sensitivity analysis included adjustment for menopausal status (pre, post) in the linear regression model.

Descriptive statistics were used to summarise patient characteristics (mean (SD) for normally distributed and median (IQR and/or range) for non-normally distributed continuous variables, and number (%) for categorical variables).

Pseudonymised data were recorded in a Microsoft Access database and statistical analysis was performed using STATA IC 13 (Stata, College Station, Texas, US).

This was an exploratory study including all available eligible patient data using the methods described. No power calculations were therefore carried out, no multiplicity testing adjustment made (33) and no replication cohort defined.

## Results

### Patient characteristics

Of 115 individuals fulfilling diagnostic criteria for hand OA during this defined time period, 92 (80%) were women and selected for further study based on fulfilling eligibility criteria (Table 1, Supplementary Figure S1). Eighty two of 92 (89%) were post-menopausal women, whilst 8 were pre-menopausal and for 2 status was unknown. 29 (31.5%) had used HRT in the past, but discontinued this, whilst 11 (12.0%) were current users and 49 (53.3%) had never used HRT (Table 1).

**Table 1:** Characteristics of study population.

Characteristics	Overall group Mean, SD or *n* (%)	Category of HRT use		
		Previous Mean, SD or *n* (%)	Current Mean, SD or *n* (%^d^)	Never Mean, SD or *n* (%)
Number (%)	92 (100)	29 (31.5)	11 (12)	49 (53.3)
Age at time of first clinic review (years)	60.3, 8.0	64.0, 7.7	59.6, 7.2	58.2, 7.8
Age at onset of hand symptoms (years)	54.8, 8.3	58.0, 9.0	52.5, 8.8	53.3, 7.2
Type of hand OA				
IPJ only	49 (53.3)	16 (55.2)	5 (45)	26 (53.1)
First CMC joint only	23 (25.0)	6 (20.7)	2 (18)	15 (30.6)
Both IPJ and first CMC joint	20 (21.7)	7 (24.1)	4 (36)	8 (16.3)
Positive family history of OA	38 (41.3)^b^	17 (58.6)	5 (46)	16 (32.7)
Nulliparous	20 (21.7)^a^	6 (20.7)	3 (27)	11 (22.4)
Post-Menopausal				
Yes	82 (89.1)	28 (96.6)	11 (100)	41 (83.7)
No	8 (8.7)	0 (0)	0 (0)	7 (14.3)
Unknown	2 (1.1)	1 (3.4)	0 (0)	1 (2.0)
Age at final menstrual period (years)	49.9, 5.2^c^	49.4 (6.6)	48.3 (4.6)	50.7 (4.2)

^a^ In 26 of 92, parity was not recorded.

^b^ In 29 of 92, family history was not recorded.

^c^ Two individuals had undergone hysterectomy/oophorectomy, of which one person had related surgical menopause. In this case, age at surgery was used as “Age at Final Menstrual Period”. HRT use was unknown for 3 individuals.

^d^ Given the low numbers (11) in this subgroup, % have been given to 2 s.f. rather than 3.

### Hand OA and menopause

**Figure 1:**
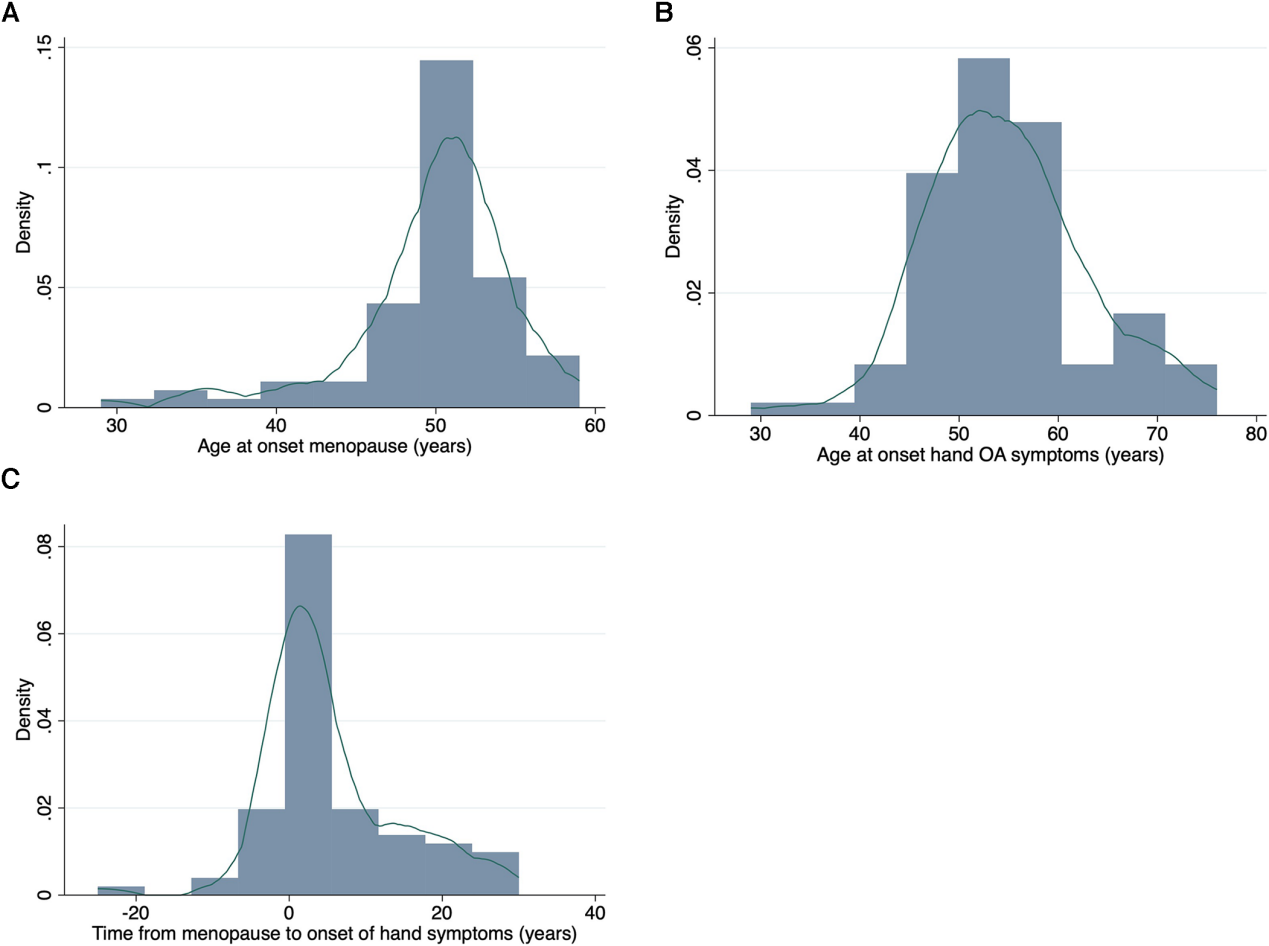
The relationship between the reported onset of menopause and hand OA symptoms. Histograms and kernel density plots of the frequency in the study population of (**A**) Age of onset of the menopause (in years) (**B**) Age of onset of hand OA symptoms (in years) and (**C**) The time between FMP (onset of menopause) and onset of hand symptoms (in years).

In these women with hand OA, hand symptom onset was at a mean of 54 years of age. The 82 post-menopausal women reported their FMP at a mean age of 49.9 years (SD 5.2) (Table 1, Figures 1A,B). In post-menopausal women, the median time from FMP to hand symptom onset was 3 years (range −25, 30 years, Figure 1C). 48/82 (59%) of these women developed their hand symptoms within the defined peri-menopausal period (FMP ± 4 years), whilst 34/82 (41%) did not.

### Hand OA and HRT use

**Figure 2:**
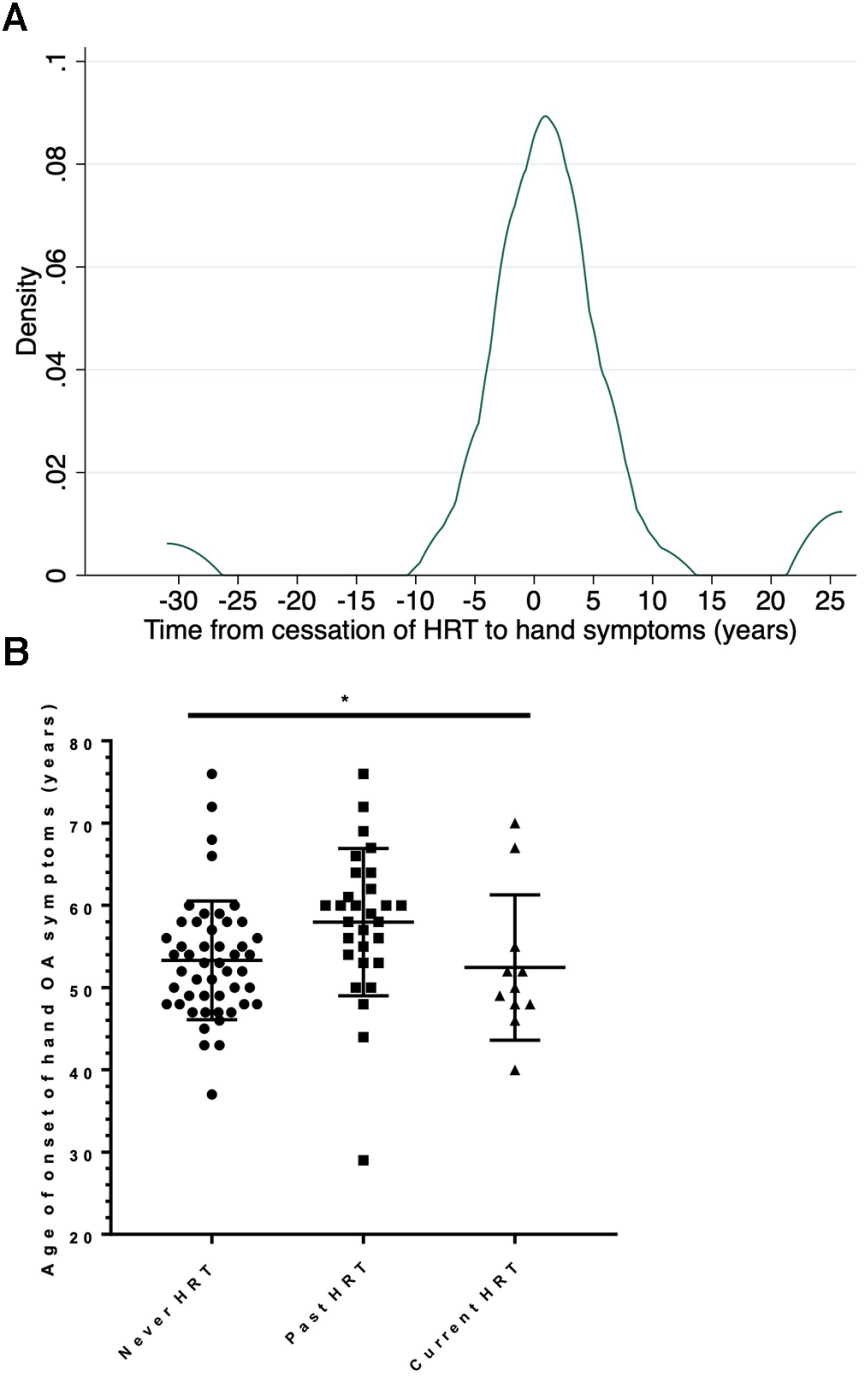
Effect of past use of HRT on the onset hand OA symptoms. (**A**) Kernel density plot of the frequency in the study population of the time between cessation of HRT and onset of symptoms in all women who were past users of HRT, *n* = 29. (**B**) The age of onset of hand symptoms is shown for 3 subgroups of postmenopausal women: those women who have never used HRT (Never HRT); those who have stopped HRT for at least 1 year (Past HRT) and those who are current users of HRT. The bar shows the mean and SD for each group. Comparison by 1 way ANOVA, *F*_2,86_ = 3.57, *P* = 0.0323.

Of those 29 women with hand OA who had used HRT but discontinued it previously, 22 (76%) had developed their hand OA after discontinuing HRT therapy and 7 (24%) between 2 and 31 years prior to that time. In these women, the median time from cessation of HRT to self-reported age at onset of hand symptoms was 6 months (Figure 2A). The age of onset of hand symptoms was compared in those who had never used HRT, current users and previous users. There was a difference in age of onset of hand symptoms between these 3 groups, with those who were previous users of HRT appearing older at symptom onset than current users or never users [mean age (SD) 58.0 (9.0), 52.5 (8.8), 53.3 (7.2) respectively for each group; ANOVA between 3 groups, *F*_2,86_ = 3.57, *P* = 0.0323], Figure 2B. Of note, most (7/11) of the women in the current HRT use group had developed hand OA symptoms prior to HRT use (so were similar to the never use group). By linear regression, when compared with the group of women who had never taken HRT, those past users of HRT were older at onset of their reported hand symptoms [coeff. 4.7 years (0.92, 8.39); *P* = 0.015], whilst those using HRT currently did not differ in age [coeff. −0.85 years (−6.17, 4.47); *P* = 0.75]. Given that those who had not taken HRT could be younger and more likely to be pre-menopausal, in an additional hypothesis test, given the null hypothesis was rejected, post-menopausal status was added as a covariate in this regression model. The association between past use of HRT and older age at onset at hand symptoms remained [coeff. 7.7 years (1.3, 14.1); *P* = 0.019].

## Discussion

In this retrospective healthcare study of women referred to a secondary care clinic for symptomatic hand OA, we report that for a substantial subgroup (but not all), there is a short interval between their menopause onset and reported onset of hand OA symptoms. Furthermore, those who had taken previous HRT appeared to develop their hand symptoms at a higher age than those who had not. However, for the majority who had taken HRT, discontinued it and developed their symptoms after stopping, they developed their symptoms within one year of cessation of therapy. Jointly, these findings lend further support to an apparent association between menopause, female hormonal change and symptomatic hand OA. Though our discussion and conclusions are necessarily cautious in this limited study which require replication in other datasets, they support the further exploration of the potential for female subtypes of hand osteoarthritis which could have clinical potential for personalising the diagnosis and treatment of this condition.

It has long been reported that hand OA increases around the age of 50, which is the typical age of menopause (11–14). However, we are not aware of any studies previously examining the temporal relationship at an individual level between onset of hand OA symptoms specifically (rather than a diagnostic code) and onset of menopause. Many OA studies do not collect data on menstrual history or menopause. Some reports from historic cohorts which have collected this information tend to focus on hand radiographic change rather than hand symptoms (23). Explanations for our findings could include menopause influencing disease susceptibility/incidence, magnifying the symptoms or severity of prevalent disease, including other related factors that enhance pain (e.g., sleep disturbance, mood) (21). It is important to emphasise that there does not appear to be a temporal relationship with menopause for all women with the condition, but rather in a substantial subgroup (3 in 5 of those studied): it is not possible to say from this study if these two subgroups clinically vary from each other in any other regard or be otherwise differentiated, which would be an important question for onwards research.

This study suggests that there may be an interaction between HRT use and onset of hand symptoms and that both starting, taking and stopping are all relevant events. These observations may partly explain the conflicting literature around HRT use and hand OA, with some studies reporting that HRT use might be protective of disease, its associated pain or radiographic disease, or delay the occurrence of symptoms (23), whilst other studies report more hand OA in HRT users or past users (18, 19). These findings are not necessarily as contradictory as they first appear: sudden cessation of HRT could be a risk factor for development of symptomatic hand OA, or its use might simply delay the onset of OA which would have occurred at some point anyway. In a recent feasibility RCT of a form of HRT, more women in the active arm developed worse symptoms than those on placebo on tapering the study medication over a 4 week period, supporting this notion of symptomatic flare on estrogen withdrawal (27). A recent review highlighted that improved advice to the subgroup of women with osteoarthritis stopping HRT could have potential practical therapeutic relevance (21). Future studies might investigate the relative severity and duration of hand symptoms in previous, current and never users of HRT.

Our data highlights the potential for treatment bias which may influence some epidemiological studies: that users of HRT may have sought HRT after onset of MSK symptoms including hand symptoms, as we saw in those with current use. This may be a reason for the apparent increased prevalence of OA in those taking HRT in some population studies (18, 19). Our findings support earlier reports from the Chingford study (a UK cohort of women) which suggested a trend towards association of HRT use and protection from radiographic distal IPJ OA (OR 0.48; 95% CI 0.17, 1.42) (symptomatic data was not reported for this cohort) (23). It also provides additional evidence to complement that from RCTs, which analyse general MSK symptoms or large joint (hip or knee) OA outcomes, but where neither symptomatic nor radiographic hand OA data were specifically collected (24, 25).

There are a number of potential mechanisms by which menopause itself and HRT use could influence the manifestation of hand OA symptoms or disease. These include changes in estrogen levels and its relative deficiency. Testosterone levels also tend to fall in menopause, and an association between low serum testosterone and presence of hand OA has been previously reported (19). Estrogen is known to fluctuate intermittently to low levels for as long as 10 years prior to menopause (29), which may be an important consideration in those developing hand symptoms several years in advance of FMP. Estrogens (principally estradiol) are produced and sensed by connective tissues including articular cartilage and bone (34). Low level estrogen replacement or its receptor modulators have been described to promote cartilage growth and new bone formation (35, 36). Estrogen is known to be anti-inflammatory and mildly immunosuppressive (20, 21). Estrogen, progesterone and testosterone also directly regulate the experience of pain (37). All of these mechanisms could be relevant to the development of symptomatic hand OA in this potential subgroup of the condition (20, 21).

Finally it is worth reflecting on the very high overall proportion of women (80%) in the sample during this period in a secondary care clinic with hand OA diagnosis. High female preponderance has been previously reported in the context of erosive OA in a secondary care setting (15). This study, which considered all forms of hand OA, raises the question of whether sex-specific phenotypes of the disease exist, which has not been particularly considered in “clinical phenotypes” to date (38).

### Limitations

It is important that in a study of this nature we do not over interpret our findings. First, this was a cross-sectional study of usually collected healthcare data: this means that no causal link can be inferred and that the apparent association between past use of HRT and increased age of hand OA symptom onset could have occurred by chance, or be influenced by unidentified or uncontrolled confounding. Second, the study relies on a retrospective recalled history of age of onset of hand symptoms and of FMP. This could be open to a lack of precision, recall bias (patient) or reporting bias (physician) if either attributed a link between the two. It is our belief that at the time of data collection (i.e., 2015 and before) there had been little publicised about any putative link, and so patients and clinicians would not have anticipated this association; also these data were systematically acquired for all patients using a standard proforma which seeks to address reporting bias.

Third, our sample size was relatively small particularly when examining pre-defined subgroups. It was not sufficient to further stratify, for example into those with IPJ or CMC joint disease only. The potential for differences (or equivalence) in these two important clinical subgroups of hand OA would be of high interest. However, combining these subgroups is frequently done in studies and trials, plus many of our patients had involvement at both sites. Similarly, it was not possible to stratify for non surgical and surgical menopause, given the very low numbers (one) in this latter group. Only 11 current users of HRT mean results relating to the association with timing of onset of hand OA symptoms in this small subgroup should be interpreted with caution.

It was not our intention to analyse further the exact nature or duration of HRT given the limitations of sample size. This could be considered in further larger studies, although it should be noted that there could be a risk of confounding unless this is controlled in some way. Available data suggest that the majority were on oral preparations of either unopposed estrogen or estrogen-progestin combinations, depending on uterine status. Some individuals had used the oral contraceptive pill (OCP) in the past. However, a systematic history of this was not routinely captured in the same way as HRT, so this could not be reported. It may be that those previously using the OCP were more likely to seek HRT than those who had not for example, and that there could be some residual confounding here with other lifetime exogenous hormone use. However, the effects of OCP use on risk of hand osteoarthritis are also not well understood.

Fourth, this study was carried out in a secondary care setting, so likely to be open to referral bias, and is therefore not necessarily generalizable to all people with hand OA symptoms. Patients in this clinic (within rheumatology outpatients) typically had a high symptom burden and disease load, often with high inflammatory symptoms in many IP joints with radiological change justifying referral. The advantage of examining such a highly symptomatic population is the associated substantial socioeconomic cost and unmet treatment need. Some people may experience more transient, or self-limiting symptoms which never reach medical review. The only possible referral routes were from primary care (family doctors) or from other MSK practitioners (hand surgeons or other rheumatologists). No self referrals or direct NHS referrals from gynaecologists or menopause clinics were possible.

Lastly, data on height was not collected at that time and therefore body mass index (BMI) was not routinely available. Increased BMI is known to affect the development of OA, including of the hand. Adipose tissue can also synthesise estrogen. In this relatively small study, sex and age have been considered but we have not attempted to adjust in a multivariable model for the influence of additional factors which could have been different between groups. In the case of BMI, individuals who were obese or have other associated comorbidities would be less likely to be prescribed oral HRT (29). Larger studies are needed to test these findings and assess their robustness and generalisability. In a larger prospective sample, one could pursue association analysis of the paired variables “age at onset of menopause” and “age at onset of hand OA symptoms” with appropriate adjustment of confounders, to understand the approximation of the distributions further.

In summary, this study reports an apparent association between the onset of menopause and timing of onset of hand OA symptoms in hand OA, which may be influenced by past use of HRT. These findings should be tested in independent larger cohorts and clinical datasets, and also examined longitudinally. Ideally these longitudinal studies would be prospective and commence prior to individuals' natural, drug or surgery induced menopause, to more fully disentangle the effects of ageing and menopause. Ultimately, only RCTs in this population may adequately control all confounding to answer important remaining questions in this area definitively.

## Data Availability

The datasets presented in this article are not readily available because the ethical permissions granted by Imperial Healthcare NHS Trust for this work did not include the sharing of datasets. Queries relating to the datasets should be directed to f.watt1@nhs.net.
