# Supplementary figures and images for: Could sex-specific subtypes of hand osteoarthritis exist? A retrospective study in women presenting to secondary care
Source: Front Pain Res (Lausanne). 2024 Feb 12;5:1331187. doi: 10.3389/fpain.2024.1331187 (PMC10895010; doi:10.3389/fpain.2024.1331187)

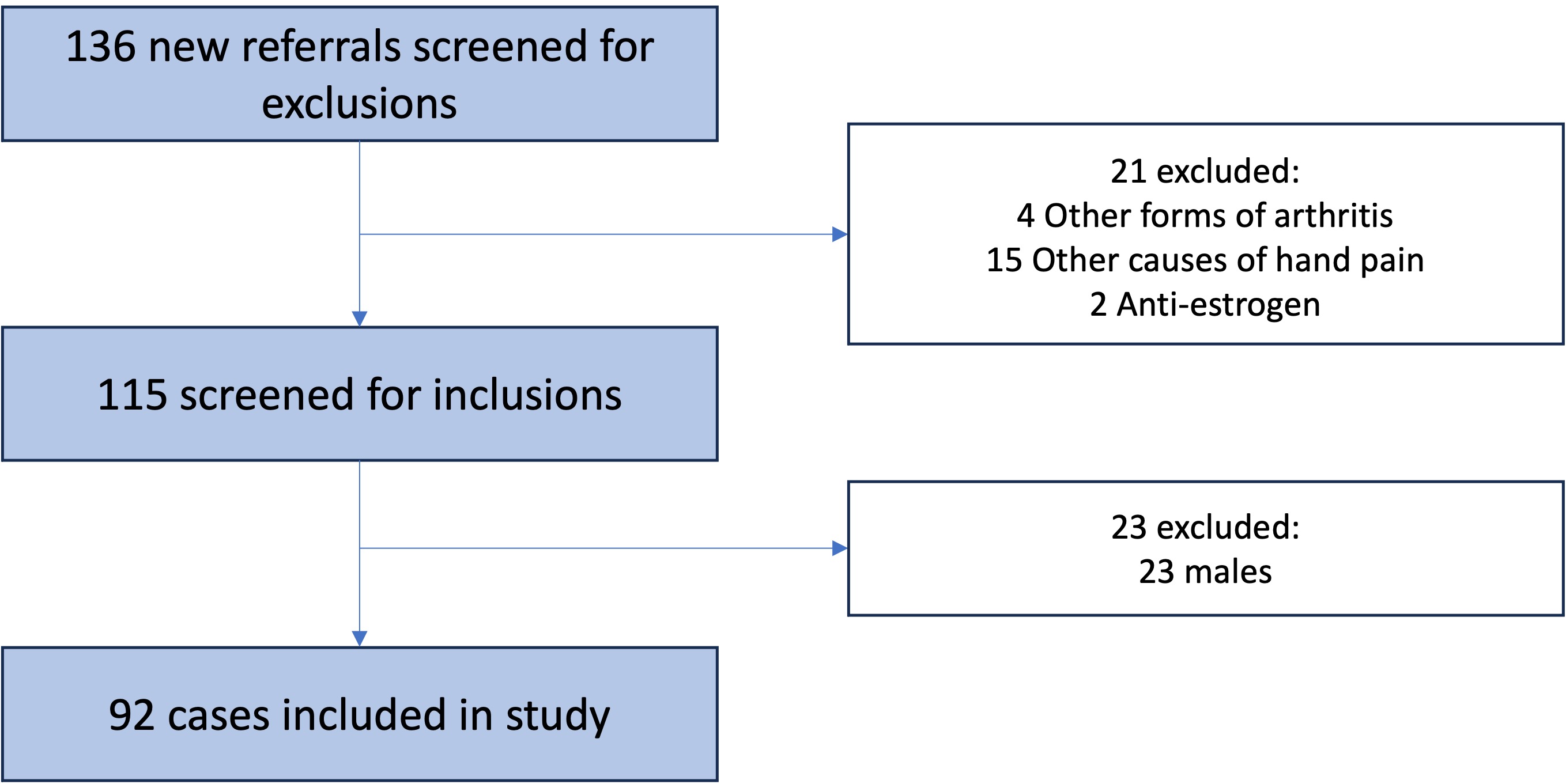

Supplement: Supplementary file 2 [file Image1.jpeg]
